# Limb Ischemia Complications of Veno-Arterial Extracorporeal Membrane Oxygenation

**DOI:** 10.3389/fmed.2022.938634

**Published:** 2022-07-15

**Authors:** Sixiong Hu, Andong Lu, Chenliang Pan, Bo Zhang, Yong ling Wa, Wenjing Qu, Ming Bai

**Affiliations:** ^1^The First School of Clinical Medicine of Lanzhou University, Lanzhou, China; ^2^Heart Center, The First Hospital of Lanzhou University, Lanzhou, China; ^3^Gansu Key Laboratory for Cardiovascular Diseases of Gansu, Lanzhou, China; ^4^Cardiovascular Clinical Research Center of Gansu, Lanzhou, China

**Keywords:** V-A ECMO, limb ischemia, risk factors, distal perfusion, salvage intervention

## Abstract

**Background:**

This study aimed to summarize and analyse the risk factors, clinical features, as well as prevention and treatment of limb ischemia complications in patients on veno-arterial extracorporeal membrane oxygenation (V-A ECMO).

**Methods:**

We retrospectively analyzed 179 adult patients who had undergone V-A ECMO support in the Cardiac Care Unit of the First Hospital of Lanzhou University between March 2019 and December 2021. Patients were divided into the limb ischemia group (LI group) and the non-limb ischemia group (nLI group) according to whether limb ischemia occurred on the ipsilateral side of femoral artery cannulation. In the LI group, patients were salvaged with a distal perfusion cannula (DPC) according to each patient's clinical conditions. The baseline data and ECMO data were compared between the two groups, and risk factors for limb ischemia complications were screened using multiple logistic regression analysis.

**Results:**

Overall, 19 patients (10.6%) had limb ischemia complications, of which 5 (2.8%) were improved after medication adjustment, 12 (8.4%) were salvaged with a DPC, and 2 had undergone surgical intervention. There were significant differences in terms of Extracorporeal Cardiopulmonary Resuscitation (ECPR), Intra-aortic balloon pump (IABP), peak vasoactive-inotropic score (VIS) within 24 h after ECMO (VIS-max), Left ventricular ejection fraction (LVEF), weaning from ECMO, and discharge rate between the two groups. ECPR, IABP, and VIS-max in the LI group were significantly higher than those in the nLI group, whereas weaning from ECMO, discharge rate, and LVEF were significantly lower in the LI group compared to those in the nLI group. Furthermore, multiple logistic regression analysis revealed that diabetes [odds ratio (OR) = 4.338, 95% confidence interval (CI): 1.193–15.772, *P* = 0.026], IABP (OR = 1.526, 95% CI: 1.038–22.026, *P* = 0.049) and VIS-max (OR = 1.054, 95% CI: 1.024–1.085, *P* < 0.001) were independent risk factors for limb ischemia complications in patients who underwent V-A ECMO.

**Conclusion:**

Diabetes, prevalence of IABP and VIS-max value in analyzed groups were independent risk factors for predicting limb ischemia complications in patients who underwent V-A ECMO. The cannulation strategy should be optimized during the establishment of V-A ECMO, and limb ischemia should be systematically evaluated after ECMO establishment. A DPC can be used as a salvage intervention for the complications of critical limb ischemia.

## Introduction

Extracorporeal membrane oxygenation (ECMO) is a mechanical cardio-respiratory support for heart and lung failure in which conventional treatment is ineffective and is increasingly becoming a bridge to permanent solution such as recovery, transplantation, more durable device, or decision. Over the past decade, the indications for ECMO have expanded, and the use of ECMO has increased exponentially (1–3). Establishing veno-arterial (V-A) ECMO by percutaneous femoral artery and venous cannulation is easy to perform, minimally invasive, and widely used in clinically. However, femoral artery cannulation may result in acute limb ischemia (ALI) of the ipsilateral limb. Severe limb ischemia can render the arterial blood supply of the lower extremities unable to meet the basic physiological and metabolic needs at rest (4–8), which may require secondary surgical intervention, or amputation and even be life-threatening (9–11). In a systematic meta-analysis by Cheng et al., the cumulative incidence of limb ischemia in ECMO-assisted patients was 16.9% (12). Therefore, detection, prevention, and management of ECMO-related ALI are critical (12–14). Many prevention strategies have been proposed to avoid limb ischemia including the selection of small arterial return cannula and cannulation side selection, cannulation technique, and placement of a smaller cannula for anterograde or retrograde (ankle) distal perfusion (15). Recently, a new bidirectional femoral arterial cannula has been proposed and tested during cardiopulmonary bypass (16). With peripheral femoral cannulation, distal perfusion ipsilateral to the femoral artery cannulation is recommended by the ELSO Red Book and guidelines (1). Currently, the placement of the distal perfusion cannula (DPC) is the most common method for preventing and treating limb ischemia complications (17, 18). However, the timing of DPC application is not clearly defined. There is a lack of clear evidence-based medical recommendations as to whether the distal perfusion catheter should be placed prophylactically or only if acute limb ischemia develops (1, 18–21).

We reviewed the data of adult V-A ECMO-assisted patients at our hospital, as well as discussed the risk factors, clinical features, and summarized the prevention and treatment of limb ischemia complications in order to improve the prognosis of these patients.

## Materials and Methods

### Study Population

We collected the clinical data of all adult patients who required V-A ECMO support between March 2019 and December 2012 in the Department of Cardiology of the First Hospital of Lanzhou University.

The inclusion criteria

(1) Patients with cardiogenic shock due to various etiologies requiring V-A ECMO assistance.
(2) Patients undergone High-risk Percutaneous Coronary Interventions (HR-PCI) who need V-A ECMO intraoperative assistance.
(3) Extracorporeal Cardiopulmonary Resuscitation (ECPR).
(4) Age between 18–80 years old.

The exclusion criteria

(1) Poor vascular access conditions, including lower extremity arterial occlusion, vascular dissection, etc.
(2) Death or weaning from ECMO due to irreversible reasons within 24 h.
(3) Patients with assistance duration <4 h.
(4) Other types of shock.
(5) Incomplete baseline data.

### Limb Ischemia

The severity of limb ischemia was graded according to the Rutherford classification (22). The amount of vasopressor should be considered and eventually reduced or discontinued after the establishment of V-A ECMO. At the same time, vasodilators are used to increase perfusion. Moreover, in the absence of bleeding, high levels of anticoagulation were maintained. DPC is placed salvagely in patients who cannot be improved by medication adjustment and in patients with Rutherford class IIb.

### Near-Infrared Spectroscopy (NIRS) Oximetry

NIRS has been used to assess limb ischemia since May 2021. The Cerebral/Somatic Oximeter (Covidien lic.,5,100 C, IRELAND) was used in all patients. The Oximeter pads for all patients were placed longitudinally on the medial aspect of the inner calf on both the cannulated and non-cannulated legs. Covidien has designated in their literature for the Cerebral/Somatic Oximeter that saturations below 40% require intervention and saturations from 40 to 50% is cautionary values for cerebral oximetry.

### V-A ECMO Management

ECMO consists of a centrifugal pump, membrane oxygenator and heparinization tube (Maquet, PLS7050/2050, Germany or LivaNava, D905, Italy), femoral arteriovenous cannula (Medtronic, Bio-Medicus, United States), and air-oxygen mixer. V-A ECMO was established by percutaneous puncture of the femoral artery (Medtronic, Bio-Medicus, 15–19 Fr cannulation) and femoral vein (19–21 Fr) under the guidance of ultrasound. Femoral arterial and venous conditions were assessed by ultrasound before ECMO cannulation. The diameter of arterial cannulation was <80% of the diameter of blood vessels.

#### Anticoagulant Management

Unfractionated heparin (80–100 U/kg) was given before cannulation, and cannulation was started after activated clotting time (ACT) of whole blood > 200 s. During the period of ECMO assistance, activated partial thromboplastin time (APTT) and D-dimer were used to monitor. APTT was monitored every 4–6 h and maintained at 40–60 S, and D-dimer was maintained without an obvious increasing trend.

#### ECMO Weaning

If the flow was reduced to <20% of the ideal cardiac output, hemodynamic stability can be maintained with low dose or without positive inotropic drugs, blood oxygen saturation≥ 95%, and echocardiography indicated that the parameters of the left ventricle and right ventricle were up to the standard and LVEF ≥ 25%, these were the criteria for considering weaning in our study.

### Distal Perfusion Cannula

For the distal perfusion cannulation, under ultrasound guidance, the superficial femoral artery was punctured in the opposite direction to the femoral artery cannulation, and an Avanti 6 F femoral sheath (Cordi, 504–606 X, Mexico) was routinely inserted and connected to the arterial end of ECMO.

We recommend the use of the “4S” scheme for the prevention and treatment of limb ischemia complications in patients on V-A ECMO.

#### Best Site Selection

The common femoral artery below the inguinal ligament and above the bifurcation is selected as the arterial puncture point.

#### Match Arterial Cannula Size

The selection of type and size of the arterial cannula should be based on a balance between the targeted flow rate and anatomical considerations, arterial cannula diameter was <80% of the diameter of blood vessels, while choosing a cannula as smaller as possible based on cardiac function.

#### Systematic Evaluation

Limb ischemia should be evaluated promptly after ECMO is established, including clinical symptoms, signs and NIRS.

#### Salvage Intervention

All patients with critical limb ischemic complications should be salvaged with a DPC placement rather than a routine placement.

### Data Collection

#### Baseline Characteristics

Age; sex; body mass index (BMI); body surface area (BSA); left ventricular ejection fraction (LVEF); relevant comorbidities, including hypertension, diabetes mellitus, coronary artery disease (CAD), and chronic respiratory disease (CRD); ECMO assistance reason, including acute myocardial infarction (AMI), acute fulminant myocarditis (AFM), extracorporeal cardiopulmonary resuscitation (ECPR), high-risk percutaneous coronary intervention (HR-PCI), and other causes. Other interventions, including intra-aortic balloon pump (IABP), continuous renal replacement therapy (CRRT), and invasive mechanical ventilation (IMV).

#### ECMO-Related Characteristics

The size of arterial cannulation; peak vasoactive-inotropic score within 24 h after ECMO established (VIS-max); ECMO duration; location of ECMO; ECMO weaning rate.

#### Outcome Indicators

Cardic Care Unit (CCU) length of stay (LOS); Hospital LOS; Successful weaning from ECMO; Discharge rate.

For calculating the vasoactive-inotropic score, all vasoactive drugs were integrated with coefficients and assigned corresponding weights, and the integrated value was used as the vasoactive drug score (vasoactive-inotropic, VIS). The calculation was as follows: VIS = dopamine + dobutamine+ 10 × milrinone + 100 × epinephrine + 100 × norepinephrine + 10,000 × vasopressin [(U / (kg ·min))].

### Statistical Analysis

All data were processed using SPSS version 24.0. Categorical and continuous variables were represented as counts (%) and medians (interquartile range). A Chi-square or Fisher's exact test was used for categorical variables, and a Student's *t*-test or Mann–Whitney U test was used for continuous variables. A multiple logistic regression analysis was used to analyze statistically significant variables to identify independent risk indicators related to neurological complications, which were summarized as odds ratios (OR) and 95% confidence intervals (95% CI). Statistical significance was set at *p* < 0.05.

## Results

### Comparison of Baseline Data

**Figure 1:**
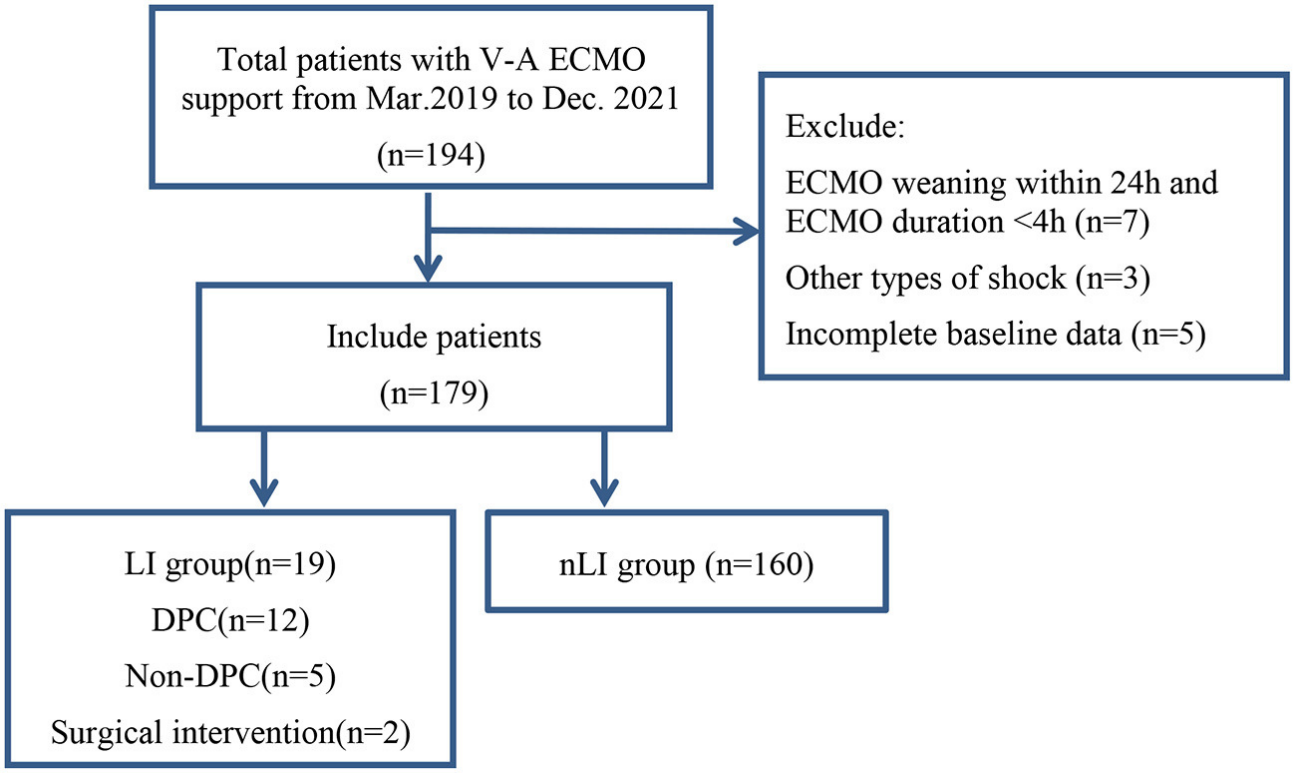
Flow chart of patient cohorts. V-A ECMO, veno-arterial extracorporeal membrane oxygenation; DPC, distal perfusion cannula; LI, limb ischemia complications group; nLI, non-limb ischemia complications group.

After excluding 15 patients, a total of 179 patients with V-A ECMO assistance were included. Patients were divided into the limb ischemia group (LI group) and a non-limb ischemia group (nLI group) based on the occurrence of limb ischemia complications (Figure 1). Among them, 146 cases were men (81.6%), 33 were women (18.4%), the average age was 59 (51, 67) years, ECMO duration was 82 (27, 120) h. 19 patients (10.6%) had limb ischemia complications, of which 5 (2.8%) improved after drug treatment, and 12 (8.4%) had salvage DPC placement. There were no statistically significant differences between the LI group and the nLI group in terms of age, sex, BMI, BSA and other basic conditions and laboratory tests. Patients were considered to be more likely to develop limb ischemia complications when the relevant comorbidities were diabetes (38.9 vs. 23.0%, *P* = 0.055), and when their etiologies for ECMO support were ECPR (27.8 vs. 11.3%, *P* < 0.046). Median LVEF values were lower in the LI group compared with the nLI group [26 (18, 23) vs. 38 (25.5, 46), *P* = 0.014]. Meanwhile, the results showed the proportion of IABP (77.8 vs. 55.6%, *P* = 0.03) was higher in the LI group (Table 1). The primary outcome indicators were CCU LOS, hospital LOS, ECMO weaning rate and discharge rate. The results showed no significant differences in CCU LOS and hospital LOS between the LI and nLI groups. However, we found that the ECMO weaning rate (50 vs. 79.5%, *P* = 0.005) and the and discharge rate (38.9 vs. 70.8%, *P* = 0.006) of the LI group were significantly lower than those of the nLI group (Table 1).

**Table 1:** Comparison of baseline characteristics of patients.

	**All patients (*n* = 179)**	**Limb ischemia**		***P***
		**Yes (*n* = 19)**	**No (*n* = 160)**	
**Baseline characteristics**	
Age (years)	59 (51,67)	60 (54.8,65)	59 (50.5,68)	0.176
Male, *n* (%)	146 (81.6)	14 (77.8)	132 (82.0)	0.66
BMI	21.8 (21.8,22.3)	26.2 (26.2,26.2)	21.8 (21.8,22.3)	0.55
BSA	1.74 (1.64,1.84)	1.65 (1.61,1.84)	1.75 (1.65,1.85)	0.39
**Relevant comorbidities**, ***n*** **(%)**	
Hypertension	72 (40.2)	9 (50)	63 (39.1)	0.372
Diabetes	44 (24.6)	7 (38.9)	37 (23.0)	0.055
CAD	150 (83.7)	16 (88.9)	134 (83.2)	0.537
CRD	10 (5.5)	2 (11.1)	8 (4.9)	0.282
**ECMO assistance reason**, ***n*** **(%)**	
AMI	116 (64.8)	15 (83.3)	101 (63.1)	0.083
AFM	21 (11.7)	1 (5.6)	20 (12.5)	0.358
ECPR	23 (12.8)	5 (27.8)	18 (11.3)	0.046^*^
HR-PCI	48 (26.8)	3 (16.7)	44 (27.5)	0.305
Other	7 (3.9)	1 (5.6)	6 (3.8)	0.743
**Laboratory test**	
AST (U/L)	38 (32, 46)	45 (36, 64)	34 (29, 51)	0.719
ALT (U/L)	33 (27, 56)	49 (31, 74)	42 (33, 58)	0.668
BUN(mmol/L)	5.54 (4.28, 7.31)	6.13 (4.79, 8.55)	5.76 (3.76, 8.21)	0.962
Hb (g/L)	95 (71, 124)	91 (69, 112.5)	98 (81, 117)	0.763
APTT (s)	86.5 (55.4, 160.2)	94.8 (62, 9, 174.6)	83.1 (54.6, 166.7)	0.882
GLU(mmol/L)	9.4 (6.5, 11.2)	9.9 (6.2, 10.8)	8.5 (7.4, 11.9)	0.365
LAC	7.3 (2.7, 8.7)	8 (4.5, 11)	6.7 (2.4, 7.7)	0.061
LVEF (%)	37 (25, 46)	26 (18, 38)	38 (25.5, 46)	0.019^*^
**Other intervenes**, ***n*** **(%)**	
IABP	103 (60.0)	14 (77.8)	89 (55.6)	0.030^*^
CRRT	51 (28.4)	7 (38.9)	44 (27.5)	0.303
IMV	58 (32.4)	9 (50)	49 (30.6)	0.093
**Outcome**	
Discharge, *n* (%)	67.5	38.9	70.8	0.006^*^
CCU LOS (days)	8 (5, 13)	8 (3, 14)	8 (6, 8)	0.704
Hospital LOS (days)	12 (8, 16)	10 (3, 19)	12 (13, 16)	0.596

*BMI, Body mass index; BSA, Body surface aera; CAD, Coronary artery disease; CRD, Chronic respiratory disease; AMI, Acute myocardial infarction; AFM, Acute fulminant myocarditis; ECPR, Extracorporeal cardiopulmonary resuscitation; HR-PCI, High risk percutaneous coronary intervention; AST, Aspartate aminotransferase; ALT, Alanine aminotransferase; BUN, blood urea nitrogen; Hb, hemoglobin; APTT, activated partial thromboplastin time; GLU, glucose; LAC, lactic acid; LVEF, Left ventricular ejection fraction; IABP, Intra-aortic balloon pump; CRRT, Continuous renal replacement therapy; IMV, Invasive mechanical ventilation; CCU, Cardiac Care Unit; LOS, length of stay*;

* *P < 0.05, the difference was statistically significant*.

### Comparison of V-A ECMO-Related Data

The locations of ECMO surgery included the cath lab (*n* = 66, 36.9%), emergency department (*n* = 51, 28.4%), and CCU (*n* = 58, 32.4%), and the median ECMO duration [106 (53, 130.9) vs. 75 (25.3, 120) days, *P* = 0.102]; the location of ECMO surgery and the ECMO duration were not significantly related to the development of limb ischemia complications. However, we found significant differences between the LI and nLI groups with respect to the median VIS-max [44 (23, 62.8) vs. 16 (10, 24), *P* = 0.014] within 24 h after ECMO established (Table 2).

**Table 2:** Comparison of V-A ECMO related characteristics.

	**All patients (*n* = 179)**	**Limb ischemia**		***P***
		**Yes (*n* = 19)**	**No (*n* = 160)**	
**Locations of ECMO**	
Cath lab	66 (36.9)	9 (47.4)	57 (35.6)	0.316
ED	51 (28.4)	0	6 (3.8)	1
CCU	58 (32.4)	10 (52.6)	97 (60.6)	0.502
**AC(F)**	17 (15,17)	17 (16.5,17)	17 (15,17)	0.094
**VISmax**	17 (10,29)	44 (23,62.8)	16 (10,25)	0.014^*^
**ECMO Duration (h)**	82 (27,120)	106 (53,130.9)	75.1 (25.3,120)	0.102
**Successful weaning from ECMO**, ***n*** **(%)**	76.5	50	79.5	0.005^*^

*ECMO, Extracorporeal membrane oxygenation; ED, Emergency department; CCU, Cardiac Care Unit; AC, Arterial cannula; VIS, Vasoactive inotropic score*;

* *P < 0.05,the difference was statistically significant*.

### Clinical Features of Limb Ischemia Complication

Among the 19 patients, 7 were treated with 15 Fr cannulation, 11 with 17 Fr cannulation, and 1 with 19 Fr cannulation; 5 cases improved after drug treatment, 12 cases were salvaged with a DPC, including 11 with a 6 F sheath and 1 was single lumen central venous catheter. One developed superficial femoral artery thrombosis and underwent interventional arterial thrombectomy and stent implantation. Femoral artery thrombosis occurred in one patient after weaning, and thrombectomy was performed. After the intervention, the lower limb ischemia gradually improved, the clinical symptoms were relieved, and there were no lower limb ulcers, gangrene, amputation, or osteofascial compartment syndrome (Table 3).

**Table 3:** Limb ischemia patients clinical features.

**Features**	**Number**	**Treatment**
ALI	5	Medication adjustment
	12	Medication adjustment + DPC
superficial femoral artery thrombus	1	embolectomy
common femoral artery thrombus	1	Incision and thrombectomy

*ALI, Acute limb ischemia; DPC, Distal perfusion cannula*.

### Multivariate Analysis of Limb Ischemia Complications in Patients Underwent V-A ECMO

Result of the univariate analysis are shown in Tables 1, 2. Baseline variables that were considered clinically relevant or that showed a univariate relationship with outcome were entered into a multiple logistic regression model. Candidate variables with a *P* value < 0.1 in univariate analysis were included in the multivariable model. The association were checked in the multivariable model, and after adjustment for ECPR, AMI, IMV, LVEF, and arterial cannula size, multivariate analysis revealed that Diabetes (OR, 4.338; 95% CI, 1.193–15.772; *P* = 0.026), IABP (OR, 1.526, 95% CI, 1.038–22.026; *P* = 0.049) and VIS-max (OR, 1.054, 95% CI, 1.024–1.085; *P < * 0.00) were independent risk factors for limb ischemia complications in patients on V-A ECMO (Table 4).

**Table 4:** Multivariate analysis of limb ischemia complications in V-A ECMO patients.

	**OR**	**95%CI**	***P***
Diabetes	4.338	1.193–15.772	0.026^*^
ECPR	1.437	0.253–8.587	0.667
AMI	1.828	0.377–8.878	0.454
IABP	1.526	1.038–22.026	0.049^*^
IMV	0.565	0.121–2.631	0.467
LVEF	0.467	0.944–1.041	0.730
AC	1.586	0.862–2.918	0.138
VIS-max	1.054	1.024–1.085	<0.001^*^

*ECPR, Extracorporeal cardiopulmonary resuscitation; AMI, Acute myocardial infarction; IABP, Intra-aortic balloon pump; IMV, Invasive mechanical ventilation; LVEF, Left ventricular ejection fraction; AC, Arterial cannula; VIS, Vasoactive inotropic score*;

* *P < 0.05, the difference was statistically significant*.

## Discussion

As a powerful life-support device, V-A ECMO is being increasingly used in clinical practice. This technique is an invasive procedure and may lead to related complications. Limb ischemia complications are often an important factor affecting the effect of ECMO. Previous studies reported that the incidence of distal limb ischemia on the ipsilateral femoral artery cannulation in patients with peripheral V-A ECMO ranges from 10 to 50% (25, 26). This may be related to differences in ECMO indications, disease distribution, baseline characteristics, implantation techniques, definition of limb ischemia, detection tools, and timing of DPC insertion. In a study of Rastan et al. (24), 5.4% of patients with femoral artery cannulation experienced limb ischemia, and the use of DPC reduced limb ischemia and fasciotomy to <40%. In a cohort study of 75 patients, the overall rate of limb ischemia was 14.7%, and the rate of limb ischemia remained 4.6% in patients treated with conventional DPC (21). Limb ischemia complications have been reported to be associated with unsuccessful ECMO weaning and have been shown to be independent predictors of in-hospital mortality (27, 28). We reviewed the incidence of limb ischemia complications, clinical features, and effectiveness of salvage DPC placement in adult patients assisted by V-A ECMO, as well as compared the effect of limb ischemia on ECMO weaning rate, discharge rate, and length of hospital stay. Our study found that the ECMO weaning and discharge rates in the LI group were significantly lower than those in the nLI group, which is consistent with previous reports in the literature. There was no significant difference in length of hospital stay between the two groups, which may be related to a higher rate of recovery and fewer serious complications. Our center is an adult heart center, with most patients with cardiogenic shock. The total incidence of limb ischemia was 10.3%, and the incidence of severe limb ischemia requiring salvage with a DPC placement was 6.5%. The incidence was relatively low, which may be related to disease distribution and treatment strategies.

ALI associated with V-A ECMO results from relative or absolute insufficiency of arterial blood flow to distal tissues, which is related to multiple factors. Larger size arterial cannulations may result in reduced lumen area and even vascular damage, resulting in limb ischemia (29). However, this relationship is not correlated, and ECMO cannula size depends on body surface area and ECMO mode, and is based on a match between target flow and vessel diameter (30). There was no significant difference in the size of arterial cannulation between the two groups in this study. Because of the lack of collateral circulation and the smaller diameter of the femoral artery, women and younger patients are prone to limb ischemia. The peripheral atherosclerotic disease also increases the risk of plaque displacement and injury during intubation and cannulation (7, 13). Diabetes and chronic respiratory disease are independent risk factors for limb ischemia during V-A ECMO (5, 31), which may be associated with chronic endothelial injury (32). In this study, the proportion of diabetes in the limb ischemia group was higher than that in the non-limb ischemia group, and multivariate analysis indicated that limb ischemia was independently associated with diabetes. Danial et al. (32) found that limb ischemia was independently associated with the Sequential Organ Failure Score (SOFA score). This also suggests that the patient's ability to compensate for hypoperfusion may affect vascular function. In addition, before the establishment of V-A ECMO, most patients are in a state of shock, requiring large doses of vasoactive drugs to maintain, especially in the ECPR state that causes vasoconstriction and acidic metabolites, which may affect distal perfusion. Our study also found that the LVEF in the ischemia group was significantly lower, the cardiac function was worse, the proportion of ECPR was higher than that in the non-ischemic group, and the VIS-max was significantly higher than that in the non-limb ischemia group. Therefore, after the establishment of ECMO, limb ischemia can be improved by drug adjustment (including reducing vasopressors, increasing vasodilators, and adequate anticoagulation) in some patients (9). For LV venting, patients with V-A ECMO-assisted cardiogenic shock often require a combined IABP, which may improve patient outcomes (33), but bilateral femoral arterial cannulation may lead to an increased risk of ALI (34). Multivariate regression analysis in this study also found that use of IABP in V-A ECMO patients was an independent risk factor for limb ischemia, and the application of dual distal perfusion catheters may be a strategy for the treatment of such patients (35).

Considering the hazards caused by limb ischemia complications, all the patient on peripheral V-A ECMO should be closely monitored, especially unconscious patients. Clinical symptoms and signs are widely used in clinical practice as the primary assessment method. The classical description of patients with limb ischemia is grouped into a mnemonic device known as the 6 Ps, including: pain, pallor, paralysis, pulseless, paraesthesia, and paralysis (36). However, these signs may be deceptive for various reasons. The continuous flow of ECMO may render patients less pulsatile and make distal pulses less detectable. In patients with cardiogenic shock, the extremities often become colder due to a high dose of inotropes and pressors despite adequate perfusion. In addition, patients with endotracheal intubation and sedation and analgesia are unable to make immediate judgments. In these cases, a more sensitive tool to detect early distal-limb ischemia is warranted. Near-infrared reflectance spectroscopy (NIRS) technology can reflect local oxygen saturation (RSO2), and tissue oxygen saturation below 50% for a duration of more than 4 min is strongly associated with hypoperfusion (37, 38). Our center is mainly for awake and non-tracheal cannula ECMO patients, and the early clinical situation is easier to monitor. However, in special populations, the combined use of NIRS to measure bilateral tissue oxygen saturation (SmO2) is helpful for early identification. The technical aspects of the various NIRS systems are not consistent. We have been using near-infrared reflectance spectroscopy as an auxiliary assessment tool for limb hypoperfusion since January 2021 but more samples are still needed to further validate its clinical relevance. Patients were also monitored to detect NIRS tissue saturation (StO2) differentials of 15% or more between the cannulated limb and the contralateral limb for the diagnosis of cannula-related obstruction to flow. The literature search for this paper found no references for intervention or cautionary values specific to distal limb saturations.

Prevention and treatment of limb ischemia should be individualized. Choosing a smaller size cannulation provides considerable clinical support and reduces complications, which is feasible in clinical practice (9, 23, 29, 39). In addition, evaluation of vascular access and selection of cannulation sites play an important role in preventing limb ischemia (1, 40–42). Proper anticoagulation, drug optimization and adequate perfusion of distal limb are necessary for patients who have occurred limb ischemia complications (9). At present, DPC is still the most commonly used invasive technique for the prevention and treatment of limb ischemia. In a meta-analysis of 1,850 patients, prophylactic DPC placement was found to reduce the incidence of limb ischemia (43). Rastan et al. (24) found that the use of DPC can reduce the risk of lower extremity ischemia and surgical intervention to <40%, so it is recommended to routinely place DPC in patients with peripheral V-A ECMO. However, there are corresponding risks associated with catheter placement, which may lead to lower extremity-related complications. In a retrospective study of 84 adult patients with V-A ECMO, Tanaka et al. found that even with prophylactic DPC placement, limb ischemic complications occurred in 12% of patients (13). Some studies suggested that DPC should be used as a salvage measure for limb ischemia after clinical assessment (21). In addition, the types of DPC used by different centers also vary greatly. The size of DPC ranges from 5 to 14 Fr and the most commonly used types are central venous catheters and vascular guide sheaths (usually 6–8 Fr) (44). In our study, we adopted the regimen of drug adjustment combined with DPC. For patients who cannot be improved by medication treatment or severe limb ischemia, DPC was placed as a salvage intervention. The limb ischemia in the whole group was improved, and no adverse events occurred, indicating DPC as a salvage intervention is feasible. Therefore, in order to prevent and treat V-A ECMO-related limb ischemia, we summarize it as a “4S” scheme (Best Site selection, Match arterial cannula Size, Systematic evaluation, Salvage intervention). During the establishment of V-A ECMO, a reasonable cannulation strategy can reduce the occurrence of limb ischemia complications to a certain extent. After ECMO was established, adequate timely evaluation and placement of DPC as a salvage intervention according to clinical conditions.

## Limitation

This retrospective case-control study yielded some meaningful results for clinical guidance. However, there are still some limitations. First, this was a single-center retrospective study, and the distribution of disease are relatively single, which may cause selection bias. Second, no more objective indicators have been recorded and NIRS was not used in all patients to assess limb ischemia. In addition, there is a lack of short-and long term follow up on prognosis. Therefore, it is necessary to conduct a randomized controlled study to confirm the effectiveness of the placement of DPC as a salvage intervention.

## Conclusion

In this study, limb ischemia complications were uncommon in patients receiving V-A ECMO assistance, but their occurrence may affect the ECMO weaning rate and survival rate. Diabetes, IABP, and VIS-max were independent risk factors for predicting limb ischemia complications in patients with V-A ECMO. It should be adequately evaluated during and after the establishment of V-A ECMO. DPC can be used as a measure of salvage for critical limb ischemic complications.

## Data Availability Statement

The raw data supporting the conclusions of this article will be made available by the authors, without undue reservation.

## Ethics Statement

The studies involving human participants were reviewed and approved by Ethics Committee of Affiliated the First Hospital of Lanzhou University. Written informed consent for participation was not required for this study in accordance with the national legislation and the institutional requirements.

## Author Contributions

SH, AL, and MB: contributed to study concept and design. YW and WQ: acquisition and analysis of data. SH and AL: writing of the original manuscript and statistical analysis. MB and AL: revision and editing of the manuscript. BZ and CP: material, administrative support, and supervision. All authors approved the final version of the manuscript and agree to be responsible for all aspects of the work.

## Funding

This work was supported by the Key Science and Technology Foundation of Gansu Province (No. 21YF5FA118) and the Natural Science Foundation of Gansu Province (No. 21JR7RA385).

## Conflict of Interest

The authors declare that the research was conducted in the absence of any commercial or financial relationships that could be construed as a potential conflict of interest.

## Publisher's Note

All claims expressed in this article are solely those of the authors and do not necessarily represent those of their affiliated organizations, or those of the publisher, the editors and the reviewers. Any product that may be evaluated in this article, or claim that may be made by its manufacturer, is not guaranteed or endorsed by the publisher.

## Abbreviations

V-A ECMO: veno-arterial extracorporeal membrane oxygenation
LI: limb ischemia
nLI: non-limb ischemia
ALI: acute Limb ischemia
CCU: cardiac care unit
DPC: distal perfusion cannula
BMI: body mass index
BSA: body surface area
CAD: coronary artery disease
CRD: chronic respiratory disease
AMI: acute myocardial infarction
AFM: acute fulminant myocarditis
ECPR: extracorporeal cardiopulmonary resuscitation
HR-PCI: high-risk percutaneous coronary intervention
AST: aspartate aminotransferase
ALT: alanine aminotransferase
BNU: blood urea nitrogen
HB: hemoglobin
APTT: activated partial thromboplastin time
GLU: glucose
LAC: lactic acid
IABP: intra-aortic balloon pump
CRRT: continuous renal replacement therapy
IMV: invasive mechanical ventilation
ED: emergency department
AC: arterial cannula
VIS: vasoactive inotropic score
LVEF: left ventricular ejection fraction
LOS: length of stay
NIRS: near-infrared spectroscopy
OR: odds ratios
CI: confidence intervals.
